# A Prospective Clinical Study of a Prosexual Nutrient: Nano Leo for Evaluation of Libido, Erection, and Orgasm in Indian Men with Erectile Dysfunction

**DOI:** 10.1155/2020/4598217

**Published:** 2020-03-11

**Authors:** S. N. Shankhwar, A. A. Mahdi, A. V. Sharma, Kishan Pv

**Affiliations:** ^1^Department of Urology, King George's Medical University, (Formerly Chhatrapati Shahuji Maharaj Medical University), Uttar Pradesh, Lucknow 226003, India; ^2^Department of Biochemistry, King George's Medical University, (Formerly Chhatrapati Shahuji Maharaj Medical University), Uttar Pradesh, Lucknow 226003, India; ^3^Regulatory Affairs and R&D, Sanzyme Pvt. Ltd., Plot No. 13, Sagar Society, Road No. 2, Banjara Hills, Hyderabad 500034, India; ^4^Head, Medical Affairs, Sanzyme Pvt Ltd, Plot No. 13, Sagar Society, Road No. 2, Banjara Hills, Hyderabad 500034, India

## Abstract

**Aim:**

The present study aimed to assess the effects of Nano Leo, a prosexual nutrient formulation, on libido, erection, and orgasm in patients with erectile dysfunction (ED).

**Methods:**

This was a prospective, single-center, phase IV efficacy study. Patients received two capsules for 7 days and thereafter one capsule through 90 days. Main outcome measures: primary endpoint was change in erectile function assessed using the International Index of Erectile Function (IIEF) questionnaire. Secondary endpoints included improvement in testosterone levels, FSH, LH, and prolactin levels; seminal parameters; and overall quality of life (QoL).

**Results:**

Our study included 99 men (mean age 32.2 ± 4.71 years). Mean erectile function domain score increased from 18.9 ± 5.67 at baseline to 23.7 ± 4.01 on day 90 (*P* < 0.001). Similar improvements were observed in orgasmic function, sexual desire, intercourse satisfaction, and overall satisfaction domains of IIEF score which was seen as early as day 30. Improved IIEF corroborated with improvement in all QoL domains. From baseline to day 90, treatment with Nano Leo increased testosterone levels (5.04 ± 2.22 vs. 5.57 ± 1.53 ng/mL, *P* < 0.001). Similar improvements were observed in orgasmic function, sexual desire, intercourse satisfaction, and overall satisfaction domains of IIEF score which was seen as early as day 30. Improved IIEF corroborated with improvement in all QoL domains. From baseline to day 90, treatment with Nano Leo increased testosterone levels (5.04 ± 2.22 vs. 5.57 ± 1.53 ng/mL,

**Conclusion:**

Nano Leo showed improved libido, erection, and orgasm as evaluated by IIEF and QoL and was well tolerated. Therefore, Nano Leo could be an effective and safe pronutrient supplement in managing ED.

## 1. Introduction

Erectile dysfunction (ED) is considered one of the most common conditions among male sexual disorders [1, 2]. It is defined as the inconsistent and recurrent inability to acquire or sustain an erection of sufficient rigidity to engage the duration of satisfactory sexual intercourse [3]. Irrespective of the definitions used or the selection of study population or sampling methods, the prevalence of ED is estimated to be 1%–10% in men aged 40–50 years and 50%–100% in those aged 70–80 years [4]. It imposes a substantial burden on male health and interpersonal relationships, including self-esteem and psychological well-being. It may also alter clinicians' belief that many psychiatric symptoms can be found among ED patients [2, 5–7]. ED is primarily a neuronal and endothelial dysfunction of the corpus cavernosum of the penis, characterized by reduced nitric oxide (NO) formation. Other etiological factors may include hypertension, androgen deficiency, atherosclerosis, high cholesterol levels, diabetes mellitus, diseases of the prostate, and anatomical deformity of the penis [8].

The present therapeutic armamentarium for ED primarily targets NO pathways, with phosphodiesterase type 5 (PDE-5) inhibition forming the first-line treatment [8, 9]. Patient discontinuation rate remains high when given PDE-5 inhibitors, and the reason can be attributed to a number of medical and psychological problems [10]. However, these drugs have certain limitations as their action may be affected by food intake and increased risk of hypotension when coadministered with alpha blockers, which can be life threatening in the case of nitrates [11]. Moreover, the efficacy of this treatment line is evident in only 60%–70% of patients, necessitating development of supplementary or alternative therapies [12].

Traditionally, in different countries with diverse cultures, various plant extracts have been evaluated for their efficacy in improving male sexual performance [13]. A majority of these have been assessed *in vitro* or have only preclinical data (e.g., *Panax ginseng*, *B. superb*, and yohimbine) [14, 15]. Hence, a need for comprehensive formulations is evident, and a combinational approach can possibly yield better results by enhancing the positive effects of such plant ingredients. Nano Leo is one such prosexual nutrient formulation containing L-arginine, *Tribulus terrestris* extract powder, *Mucuna pruriens*, *Ginkgo biloba*, zinc as zinc monohydrate, and Yohimbe bark extract. All of these active constituents have shown to improve sexual dysfunction [16–22]. The present study aimed to assess the effects of Nano Leo treatment on libido, erection, and orgasm in patients with ED.

## 2. Materials and Methods

### 2.1. Study Design and Patient Selection

This was a prospective, single-center, phase IV efficacy study. The study has been registered at the Clinical Trials Registry India (Registration No: CTRI/Ref/2011/10/002895) and has been approved by ethics committee, King George Medical College, Lucknow. The study was conducted at outpatient clinics of participating urologist from November 2011 to April 2012 (6 months). The study was performed in accordance with ethical principles of Declaration of Helsinki, International Conference on Harmonisation (ICH)-Good Clinical Practice (GCP) guidelines, and an IEC-approved protocol. All patients provided informed consent before participation in the study. This study included Asian origin men aged 22 to 44 years with a stable sexual partnership during the previous 6 months and mild to moderate ED, as judged by IIEF scores of <20. Patients were excluded from the study if they had congenital penile disorder, cryptorchidism (absence of both testes), sperm cord disorder, varicocele, sexually transmitted diseases (STDs), infective liver disorder, azoospermia, or aspermia.

### 2.2. Study Procedure

The study drug Nano Leo, as soft gelatin (SG) capsules, was dispensed to all patients as blister packs of 15 for oral administration. Each 1.4 gm capsule contained L-arginine 500 mg, *Tribulus terrestris* extract powder 200 mg, Yohimbe bark extract 1 mg, *Mucuna pruriens* 20 mg, *Gingko biloba* 20 mg, and zinc as zinc monohydrate 20 mg. At initiation, a loading dose of two SG capsules was administered at bedtime for 7 days, followed by one SG capsule administered every day at bedtime for 90 days. However, no placebo was given to the patients. The study included 3 follow-up visits on days 30, 60, and 90. All patients were provided a calendar sheet (along with a calendar card) for ease of administration and improved trackability. Physical examination, vital signs, prior and current concomitant medications, compliance to study medications, and adverse events (AEs) were recorded at all visits. Efficacy measures were assessed at baseline and at all follow-up visits.

### 2.3. Outcomes and Endpoints

The effect of study formulation on sexual functioning was evaluated using an IIEF questionnaire. This questionnaire is a validated, self-reported tool for assessing ED and measuring treatment response [23]. It includes various facets of sexual behavior categorized into five domains: erectile function (EF), sexual desire, orgasmic function (OF), intercourse satisfaction (IS), and overall satisfaction (OS). The primary endpoint was a change from baseline to 90 days in IIEF scores for overall improvement in EF, libido, and orgasm. Secondary endpoints included improvement in testosterone levels; seminal parameters; FSH, LH, and prolactin levels; and overall quality of life (QoL) from baseline to end of the study period.

### 2.4. Statistical Analyses

Continuous variables are presented as mean values and standard deviations (SD). Nominal variables are presented as number of observations (N) and percentages (%). For continuous variables, comparisons between visits were performed using one-way analysis of variance (ANOVA) followed by the Bonferroni test or the Friedman test. In addition, pairwise comparisons of values between visits were performed using Student's *t*-test or the Wilcoxon signed rank test. A *P* value of ≤0.05 was considered statistically significant. SPSS version 14.0 (SPSS Inc., Chicago, IL) was used for all statistical analyses.

## 3. Results

Table 1 summarizes demographic and baseline clinical characteristics. In brief, our study included 99 men aged 22–44 (32.2 ± 4.71) years; among them, 29 (29.3%) patients were smokers, 69 (69.7%) were nonsmokers, and only 1 (1.0%) was an ex-smoker. Similarly, 12 (12.1%) patients were alcoholic, 86 (86.9%) were nonalcoholic, and only 1 (1.0%) was an ex-alcoholic (Table 1). Analyses of initial medical examination revealed that none of the patients enrolled had severe metabolic disorder, cardiovascular diseases, carcinoma of testis, hypothalamus or pituitary, congenital abnormalities in penis or testis, Kallmann's, Klinefelter's, and Y-deletion disorders, obesity, and type 2 diabetes mellitus. Mean EF domain scores significantly increased from 18.9 ± 5.67 at baseline to 23.7 ± 4.01 at 90 days (*P* < 0.001), and this improvement was seen as early as in 30 days. Moreover, similar significant improvements from baseline were noted in other domains such as OF, SD, and OS (add all) on days 30, 60, and 90, indicating overall improvement in symptoms of ED by Nano Leo (Table 2).  Table 3 presents a summary of responses from baseline to last follow-up visits to the QoL and general well-being questionnaire. Responses indicated overall improvement in QoL in all QoL domains from baseline to 90 days. By visit 4 at 90 days, 51% (50) of patients reported their general well-being to be very good. Moreover, 52% of patients reported improvement in their overall mental/emotional state as very good by 90 days. Furthermore, patients' overall ability to handle pressure was very good as it increased up to 73.6% by visit 4 at 90 days. By visit 4, approximately 88.7% and 95.8% of patients rated their overall enjoyment of life and their overall QoL, respectively, to be in the range of good to excellent.

On the contrary, treatment with Nano Leo showed significantly improved testosterone levels (Table 4). The mean (SD) value at visits 1 and 4 was 5.04 ± 2.22 and 5.57 ± 1.53 ng/mL, respectively (*P* < 0.05). In addition, prolactin levels increased from 211.58 ± 70.01 *μ*IU/mL at baseline to 217.99 ± 71.00 *μ*IU/mL at 90 days; this increase was correlated with corresponding reduction in FSH and LH levels (7.12 ± 5.68 to 7.01 ± 4.23 mIU/mL and 6.51 ± 4.20 to 6.48 ± 3.42 mIU/mL, respectively; *P* > 0.05) by 90 days (Table 4). However, these changes did not reach statistical significance.

Table 5 enlists the sperm characteristics assessed based on WHO guidelines. By day 90, the sperm concentration significantly increased from 44.07 ± 48.28 (baseline) to 56.21 ± 50.45 million/mL and the total sperm count per ejaculate significantly increased from 130.40 ± 156.05 (baseline) to 142.5 ± 161.23 million/mL. Total motile spermatozoa increased from 47.22 ± 55.11 million/mL to 55.27 ± 64.59 million/mL at 90 days.

Analysis of vital signs, physical examination, liver function test, and renal function test indicated no abnormal findings.

## 4. Discussion

The present study evaluated potential benefits of Nano Leo formulation in improving ED, orgasm, and libido. Ninety days of treatment with Nano Leo significantly improved all IIEF domains scores. Moreover, the treatment resulted in increase in testosterone levels and sperm count. Our results indicate that Nano Leo may provide benefits in terms of improving/enhancing the overall quality of sexual experience in men.

Men are usually reluctant to visit physicians and discuss their sexual problems, particularly for conditions such as ED; hence, typically, they frequently rely on herbal formulations prepared using complementary and alternative medicines [24]. Traditional medicine practitioners believe that multiherb supplements can improve efficacy and reduce adverse effects [25]. These combinations aim to achieve net additive or synergistic effects of its individual ingredients with similar clinical/pharmacological actions. However, efficacy of such formulations has not been adequately supported by conclusive clinical study reports. Nano Leo is a combination of L-arginine and several herbs. Results from the present study provide proof of clinical benefits with Nano Leo treatment, which is a combinatorial formulation of L-arginine with several herbs.

The hypothesis that high doses of L-arginine may provide e-NOS with abundant substrate that could result in enhanced NO formation and mitigate ED symptoms has been tested in several clinical trials. In a randomized, placebo-controlled study with L-arginine at 3 × 500 mg/day, patients reported significant improvement in erectile function at the end of the study period [16]. In addition, aphrodisiac properties of *Tribulus* have been demonstrated in animal models as well as in a clinical study [17]. It was postulated that yohimbine may exert synergistic benefits when administered with L-arginine [18]. A double-blind, placebo-controlled, three-way crossover study conducted using a combination of yohimbine and L-arginine showed that yohimbine boosts erectile function by improving blood flow and that administration of this combination was effective in improving erectile function in patients with mild to moderate ED [18]. *M. pruriens*, a tropical legume, has shown to improve steroidogenesis and semen quality in infertile men [19]. A study on human cell lines showed that use of water fraction of *M. pruriens* seed extracts was more potent than the positive control sildenafil at upregulating nNOS gene expression in neurons, indicating its use in ED [18]. Another active constituent of our product is *Ginkgo biloba* extract, which has clinically shown to improve sexual dysfunction, and its beneficial effects have been attributed to increase in neuronal nitric oxide synthase (nNOS) levels [21]. Similarly, zinc therapy has demonstrated to improve level of sexual functions [22]. In agreement with these results, it can be suggested that each active constituent of Nano Leo may be contributing to its beneficial effects in alleviating ED symptoms.

ED has been thought to be a disease of ageing men; however, several studies have shown that this condition is a major health concern among young men as well [26]. Consistent with previous reports, the mean age of study participants in our study was 32.2 ± 4.71 years. Moreover, ED in young men has different symptomology: they show lower relationship satisfaction, more depressive behavior, more negative reactions from partners, and less job satisfaction [27]. ED poses a psychological threat to the patients and can be associated with generalized anxiety disorder, panic-attack, or social phobia and these may alter clinician's perspective while evaluating patients with ED. Although, psychiatric symptoms and mental disorders are often comorbid with impaired sexual response, only few studies have specifically addressed its association with ED [7].

Young men perceive ED as a serious disability that adversely affects their active and perfect sexual life in contrast to old men who find ED to be a normal and irreversible ageing process. These behavioral aspects might prompt such individuals to seek medical help more often compared with their older counterparts. Our results indicate that Nano Leo is effective in improving ED symptoms in younger men, as reflected by improvement in IIEF scores and QoL responses.

Several trials evaluating patient preferences or treatment-switching patterns among available PDE-5 inhibitors, the first-line treatment for ED, have indicated a growing importance of patient choice in clinical decision-making. Despite higher efficacy, good tolerability, and ease of administration, long-term treatment adherence with PDE-5 inhibitors is poor [28]. A meta-analysis of 162,936 patients (mean age: 58.8 ± 7.9 years) reported PDE-5 inhibitors were associated with higher discontinuation rate (almost 50% after 1 year) mostly in younger subjects. Partner-related problems and efficacy were the main reasons of PDE-5 inhibitor dropout. No single factor played a major role in dropout, suggesting that discontinuation rate is attributable to a combination of medical problems and psychosocial and relational factors [10]. Our study primarily focused on crystalizing patients' perspective based on their responses to patient-reported measures such as IIEF and QoL. The results established improvements in sexual function, recorded by IIEF and as corroborated by a significantly greater proportion of patients responding to better grades in the QoL questionnaire.

Treatment with Nano Leo resulted in increase in testosterone levels. Such increase has been observed in previous studies wherein plant extracts were evaluated [13, 29]. A possible explanation was that although the extracts do not directly stimulate testosterone synthesis, it is mediated via increased intercourse activity due to enhanced erectile capability. The increased testosterone levels lead to its higher conversion into estradiol that directly affects libido [29].

Of note, the study drug did not have any adverse effects at the prescribed dosage and neither on blood pressure parameters nor on hepatic and renal profiles. Moreover, no AEs were observed on laboratory examination or vital measurements.

A major limitation of our study was that none of the patients were randomized, our study drug was administered to all patients, and no placebo was given during the entire study duration. Furthermore, our study did not enroll patients with other comorbid conditions such as diabetes or hypertension, which are frequently associated with ED. Moreover, this was a single-center study; hence, this limits the generalization of data for patients with other concurrent diseases and medications and cannot be extrapolated to other ethnicities or geographies.

## 5. Conclusions

In summary, Nano Leo treatment significantly improved IIEF scores and QoL. It was well tolerated and safe and could be useful to mitigate symptoms of ED and improve erectile function in men. However, these findings need to be substantiated in well-designed studies.

## Figures and Tables

**Table 1:** Demographic and baseline clinical characteristics.

Baseline characteristics	
Age, years	32.2 ± 4.71
Smoking (%)	
Yes	29 (29.3)
Ex-smoker	1 (1)
Alcohol use (%)	
Yes	12 (12.1)
Ex-alcoholic	1 (1)
BMI, kg/m^2^	23.98 (2.46)
Heart rate, BPM	73.8 (3.93)
SBP (supine), mmHg	124.3 (8.07)
DBP (supine), mmHg	80.4 (5.70)
SBP (sitting), mmHg	123.7 (7.64)
DBP (sitting), mmHg	79.9 (5.43)

Data are shown as mean ± SD or *N* (%).

**Table 2:** Distribution of patients in each IIEF domain and mean domain scores.

Domain	Duration	Domain categories					Mean score (mean ± SD)
		Severe (%)	Moderate (%)	Mild to moderate (%)	Mild (%)	No (%)		
Erectile function	*V*1	5 (5.1)	10 (10.1)	27 (27.3)	41 (41.4)	16 (16.2)	18.9 ± 5.67
	*V*2	1 (1)	5 (5.1)	23 (23.5)	43 (43.9)	26 (26.5)	21.0 ± 4.96^*∗*^
	*V*3	0	2 (2)	15 (15.3)	42 (42.9)	39 (39.8)	22.7 ± 4.1^*∗*^
	*V*4	0	3 (3.1)	7 (7.1)	35 (35.7)	53 (54.1)	23.7 ± 4.01^*∗*^

Orgasmic function	*V*1	4 (4)	12 (12.1)	30 (30.3)	34 (34.3)	19 (19.2)	6.6 ± 2
	*V*2	0	7 (7.1)	22 (22.4)	47 (48)	22 (22.4)	7.2 ± 1.59^*∗*^
	*V*3	0	7 (7.1)	14 (14.3)	52 (53.1)	25 (25.5)	7.5 ± 1.55^*∗*^
	*V*4	0	5 (5.1)	7 (7.1)	51 (52)	35 (35.7)	7.9 ± 1.38^*∗*^

Sexual desire	*V*1	2 (2)	21 (21.2)	52 (52.5)	23 (23.2)	1 (1)	5.7 ± 1.46
	*V*2	0	11 (11.2)	50 (51)	35 (35.7)	2 (2)	6.2 ± 1.38^*∗*^
	*V*3	0	5 (5.1)	37 (37.8)	51 (52)	5 (5.1)	6.8 ± 1.36^*∗*^
	*V*4	0	4 (4.1)	23 (23.5)	64 (65.3)	7 (7.1)	7.2 ± 1.34^*∗*^

Intercourse satisfaction	*V*1	4 (4)	19 (19.2)	30 (30.3)	31 (31.3)	15 (15.2)	9.1 ± 3.05
	*V*2	3 (3.1)	10 (10.2)	26 (26.5)	46 (46.9)	13 (13.3)	9.9 ± 2.72^$^
	*V*3	0	6 (6.1)	22 (22.4)	34 (34.7)	36 (36.7)	11 ± 2.64^*∗*^
	*V*4	0	6 (6.1)	11 (11.2)	39 (39.8)	42 (42.9)	11.5 ± 2.51^*∗*^

Overall satisfaction	*V*1	8 (8.1)	21 (21.2)	16 (16.2)	26 (26.3)	28 (28.3)	6.7 ± 2.5
	*V*2	2 (2)	14 (14.3)	15 (15.3)	30 (30.6)	37 (37.8)	7.4 ± 2.14^*∗*^
	*V*3	0	7 (7.1)	15 (15.3)	32 (32.7)	44 (44.9)	8.2 ± 1.9^*∗*^
	*V*4	0	3 (3.1)	13 (13.3)	29 (29.6)	53 (54.1)	8.6 ± 1.67^*∗*^

*V*1: baseline, *V*2: at 30 days, *V*3: at 60 days, and *V*4: at 90 days; ^*∗*^*P* < 0.001 and ^$^*P* < 0.0002 from Wilcoxon signed rank test; *P* < 0.001 from Friedman test for overall comparisons.

**Table 3:** Distribution of patients in each QoL domain.

Domain	Status	Patients, *n* (%)			
		*V*1 (%)	*V*2 (%)	*V*3 (%)	*V*4 (%)
Overall physical well-being	Excellent	9 (9.1)	3 (3.1)	5 (5.1)	9 (9.2%)
	Very good	33 (33.3)	53 (54.1)	54 (55.1)	50 (51.0)
	Good	51 (51.5)	41 (41.8)	39 (39.8)	38 (38.8)
	Fair	6 (6.1)	1 (1.0)	0	1 (1.0)
	Poor	0	0	0	0

Overall mental/emotional state	Excellent	2 (2.0)	1 (1.0)	2 (2.0)	6 (6.1)
	Very good	31 (31.3)	39 (39.8)	43 (43.9)	51 (52.0)
	Good	54 (54.5)	54 (55.1)	52 (53.1)	40 (40.8)
	Fair	12 (12.1)	4 (4.1)	1 (1.0)	1 (1.0)
	Poor	0	0	0	0

Overall ability to handle stress	Excellent	2 (2.0)	3 (3.1)	1 (1.0)	5 (5.1)
	Very good	18 (18.2)	19 (19.4)	22 (22.4)	31 (31.6)
	Good	68 (68.7)	66 (67.3)	68 (69.4)	56 (57.1)
	Fair	11 (11.1)	10 (10.2)	7 (7.1)	6 (6.1)
	Poor	0	0	0	0

Overall enjoyment of life	Excellent	2 (2.0)	6 (6.1)	2 (2.0)	7 (7.1)
	Very good	13 (13.1)	17 (17.3)	24 (24.5)	27 (27.6)
	Good	58 (58.6)	57 (58.2)	58 (59.2)	54 (55.1)
	Fair	25 (25.3)	18 (18.4)	14 (14.3)	10 (10.2)
	Poor	1 (1.0)	0	0	0

Overall quality of life	Excellent	1 (1.0)	4 (4.1)	2 (2.0)	7 (7.1)
	Very good	15 (15.2)	15 (15.3)	26 (26.5)	31 (31.6)
	Good	71 (71.7)	70 (71.4)	66 (67.3)	56 (57.1)
	Fair	12 (12.1)	9 (9.2)	4 (4.1)	4 (4.1)
	Poor	0	0	0	0

*V*1: baseline, *V*2: at 30 days, *V*3: at 60 days, and *V*4: at 90 days.

**Table 4:** Changes in hormone levels.

Lab parameters	Mean ± SD
Testosterone (ng/mL)	
V1	5.04 ± 2.22
V4	5.57 ± 1.53^*∗*^
Change from baseline	−0.52 ± 1.93
FSH levels (mIU/mL)	
V1	7.12 ± 5.68
V4	7.01 ± 4.23
Change from baseline	0.12 ± 4.23
LH levels (mIU/mL)	
V1	6.51 ± 4.20
V4	6.48 ± 3.42
Change from baseline	0.03 ± 2.62
Prolactin (*μ*IU/mL)	
V1	211.58 ± 70.01
V4	217.99 ± 71.00
Change from baseline	−6.98 ± 84.36

FSH: follicle stimulating hormone; LH: luteinizing hormone; *V*1: baseline, *V*2: at 30 days, *V*3: at 60 days, and *V*4: at 90 days; ^*∗*^*P* < 0.05.

**Table 5:** Sperm parameters.

Parameter	*V*1 (*n* = 99)	*V*2 (*n* = 98)	*V*3 (*n* = 98)	*V*4 (*n* = 98)	*P* value
Negative fructose level	99 (100.0)	97 (99.0)	98 (100.0)	98 (100.0)	
Alkaline pH	99 (100.0)	97 (99.0)	98 (100.0)	98 (100.0)	
Sperm concentration (million/mL)	44.07 ± 48.28	48.22 ± 47.15^*∗*^	45.73 ± 46.65	56.21 ± 50.45^*∗*^	<0.0001^*∗∗*^
Total sperm count per ejaculate (million)	130.40 ± 156.05	137.74 (143.28)^*∗*^	136.02 (134.61)^*∗*^	142.50 (161.23)^*∗*^	<0.0001^*∗∗*^
Motility (%)	45.6 (23.06)	46.2 (20.67)	46.9 (19.16)	47.2 (18.97)	0.0015^*∗∗*^
Morphology (%)	26.1 (17.43)	25.1 (15.54)	25.2 (14.35)	25.0 (12.64)	0.4656^*∗∗*^
Volume (mL)	3.01 (0.892)	3.49 (3.820)	3.57 (3.775)	3.14 (0.761)	0.1256^*∗∗*^
Liquefaction (minutes)	25.6 (6.05)	26.6 (5.72)	26.0 (5.19)	26.2 (5.62)	0.2552^*∗∗*^
Total motile spermatozoa	47.22 (55.11)	37.33 (17.55)^*∗*^	39.23 (17.62)^*∗*^	55.27 (64.59)^*∗*^	<0.0001^*∗∗*^

*V*1: baseline, *V*2: at 30 days, *V*3: at 60 days, and *V*4: at 90 days; ^*∗*^*P* < 0.05 vs. *V*1; ^*∗∗*^*P* < 0.001 from Friedman test for overall comparisons; data are shown as mean ± SD or *n* (%).

## Data Availability

The data used to support the findings of this study are included within the article.
